# Novel methods to optimize gene and statistic test for evaluation – an application for *Escherichia coli*

**DOI:** 10.1186/s12859-017-1517-z

**Published:** 2017-02-10

**Authors:** Tran Tuan-Anh, Le Thi Ly, Ngo Quoc Viet, Pham The Bao

**Affiliations:** 1 Faculty of Mathematics and Computer Science, VNUHCM-University of Science, 227 Nguyen Van Cu Street, District 5, Ho Chi Minh City, Vietnam; 2 School of Biotechnology, VNUHCM-International University, Quarter 6, Linh Trung Ward, Thu Duc District, Ho Chi Minh City, Vietnam; 3 Faculty of Information Technology, Ho Chi Minh City University of Pedagogy, 280 An Duong Vuong Street, Ward 4, District 5, Ho Chi Minh City, Vietnam

**Keywords:** Gene optimization, Neural network, Bayes’ theorem, Euclidean distance, Codon usage bias, Highly expressed gene

## Abstract

**Background:**

Since the recombinant protein was discovered, it has become more popular in many aspects of life science. The value of global pharmaceutical market was $87 billion in 2008 and the sales for industrial enzyme exceeded $4 billion in 2012. This is strong evidence showing the great potential of recombinant protein. However, native genes introduced into a host can cause incompatibility of codon usage bias, GC content, repeat region, Shine-Dalgarno sequence with host’s expression system, so the yields can fall down significantly. Hence, we propose novel methods for gene optimization based on neural network, Bayesian theory, and Euclidian distance.

**Result:**

The correlation coefficients of our neural network are 0.86, 0.73, and 0.90 in training, validation, and testing process. In addition, genes optimized by our methods seem to associate with highly expressed genes and give reasonable codon adaptation index values. Furthermore, genes optimized by the proposed methods are highly matched with the previous experimental data.

**Conclusion:**

The proposed methods have high potential for gene optimization and further researches in gene expression. We built a demonstrative program using Matlab R2014a under Mac OS X. The program was published in both standalone executable program and Matlab function files. The developed program can be accessed from http://www.math.hcmus.edu.vn/~ptbao/paper_soft/GeneOptProg/.

## Background

Since Paul Berg and Peter Lobban each independently proposed an approach to generate recombinant DNA in 1969 – 1970, recombinant protein has become a widespread tool for both cellular and molecular biology. For instance, in 2004, more than 75 recombinant proteins were used as medicine and more than 360 pharmaceuticals based on recombinant protein were under development [1]. Moreover, Elena’s study indicated that the global market of industrial enzymes exceeded $4 billion in 2012 [2]. In the future, this figure can be raised considerably thanks to the applications of synthetic biology tools which will improve the productivity of recombinant proteins production.

The increment in recombinant protein productivity reduces a significant production cost, so it might dramatically raise profits. In order to improve the productivity, several aspects can be optimized such as purification process, culture medium and genetic materials (including operator, promoter, and gene). In this study, we only focus on gene optimization.

Introducing native genes into a host can cause incompatibility of codon usage bias, GC content, repeat region, Shine-Dalgarno sequence with host’s expression system. The yields can fall down significantly [3–7]. In a culture medium, synonymous genes, which share the same operator and promoter, can be expressed at different levels. The synthetic codon optimized gene results in protein level that were ~2 to 22 fold greater than the amounts reported for the native genes [8–12]. A gene optimization program based on machine learning approach and experimental data can handle redesign task rapidly instead of using “brute force” method, which consume more significant times than other resources.

The foundation of gene optimization is a phenomenon of codon synonym. A codon is constructed by three ribonucleotides, so there are 4^3^ = 64 codons (there are 4 kinds of ribonucleotides: A – Adenine, U – Uracil, G – Guanine and C - Cytosine). However, 61 types of codons can code for only 20 kinds of amino acids. This means there must be several amino acids encoded by at least two codons. If one amino acid is coded by several codons, these codons are called synonymous codons. Moreover, codon usage is diverse from organism to organism [3, 13–15]. Generally, genes having compatible codon usage bias with host’s expression system are usually highly expressed in translational levels.

The aim of gene optimization program is to indicate which synonymous genes can give higher yield by using variety of approach including one amino acid – one codon (JCAT, Codon Optimizer, INCA, UPGene, Gene Designer), randomization (DNA Works), hybrid (DNA Works), Monte Carlo (Gene Designer), genetic algorithm (GASSCO, EuGene), etc. [16–22]. In some case, one amino acid – one codon method which replaces rare codons by the most preferable usage codons can result in worse protein expression as reported in many past studies [11, 23–26]. Yields of genes redesigned by randomization method are greater than yields of native genes, yet the result is uncertain and we cannot predict expression level until experiment finished [11, 20]. Genetic algorithm and Monte Carlo method with linear target function seem more reasonable than other reported methods. However, parameter estimation has been yet reported [18, 27]. A nonlinear method based on neural network was proposed but an analysis of its performance was not provided [28]. Some redesigned genes were proven for high expression by experiment [19, 29–31]. However, the most important disadvantage is that almost all of these studies did not provide any method to construct the model within an actual experimental data and to evaluate the optimization methods based on statistics [16–20, 22, 27].

Machine learning approaches have been developed rapidly for recent decades. These methods could analyze and “learn” pattern from data sources and predict precisely the outcome of a new data instance. Artificial neural network (NN) and Bayesian decision are two of the most efficient and popular machine learning algorithm worldwide. NN is a strong learning technique and appropriated with both regression and classification problem. Bayesian decision is highly acclaimed due to its simplicity.

These are the reason why we propose a novel method for gene optimization base on Bayesian theory and Neural network which are the most common learning methods using probability and statistics background. We also use statistic test to evaluate and compare these methods.

## Methods

### Data collection

We used highly expressed genes (HEG) as the reference set for codon adaptation index (CAI) computing [32]. We also collected redesigned genes and respective translational expression levels of product (Table 1). The experimental data collection process was based on four criteria: 1) expression system should be *Escherichia coli*, 2) the experiments should express both native and optimized genes, 3) the sequences and respective quantitative productivity should be provided, and 4) expression level should be recorded or could be converted to mg/L. The data would be used to form an NN in a later step.

**Table 1:** Collected data including redesigned genes and respective product

Host	Product	Number of genes	Reference
E. coli BL21	DNA Polymerase and scFV	62	[46]
E. coli BL21	Cystatin C	2	[12]
E. coli BL21	PEDF	2	[9]
E. coli W3110	Prochymosin	7	[11]

### Codon usage bias measurements

The preference of codons is correlated with intracellular tRNA concentration in a host environment and reflects a balance necessary for expression efficiency [3, 6, 9, 15]. Translation process can be delayed when ribosomes encounter rare codons, which can cause ribosomes to detach from mRNA and abort translation [9]. Moreover, mRNA translation rate may impact the secondary structure of encoded protein in that frequently used codons tend to encode for structural elements while rare codons are associated with linkers [11, 33, 34].

CAI is one of the most popular and effective measures for quantifying codon usage bias of a gene toward a reference set of highly expressed genes [35]. Given a gene *g* = {*g*_1_, *g*_2_, …, *g*_*i*_, … *g*_*L*(*g*)_}, CAI is defined as (1)

1 $$ C A I(g)={\left({\displaystyle \prod_{i=1}^{L\ (g)}{w}_{a{ g}_i}}\right)}^{\frac{1}{L(g)}} $$

where *L*(*g*) is the length of gene *g* counted by codon, *g*_*i*_ is the *i* ^th^ codon of gene *g*, *ac* is generally a codon *c* coding for amino acid *a*. In this case, *c*≡*g*_*i*_, *w*_*ac*_ described as (2) is the relative adaptiveness of *ac*, and *o*_*ac*_(*HEG*) is the count of *ac* in HEG set.

2 $$ {w}_{ac}=\frac{o_{ac}(HEG)}{\underset{o_{ac}}{ \max }{o}_{ac}(HEG)} $$

Relative synonymous codon usage (RSCU), which maps genes into a 59-dimensional vector space is also a common measure and widely used in gene clustering [36]. The RSCU is

3 $$ {r}_{a c}(g)=\frac{o_{a c}(g)}{\frac{1}{k_a}{\displaystyle {\sum}_{c\in {C}_a}}{o}_{a c}(g)} $$

where *C*_*a*_ = {*ac*|*ac is the codon c coding for amino acid a*}, and *k*_*a*_ = |*C*_*a*_|.

### GC content

Some studies indicated that GC content can impact the stability of the 2^nd^ structure of mRNA which was beneficial for translation [7, 10]. GC content is computed as (4)

4 $$ G C(g)=\frac{o_{GC}(g)}{L(g)} $$

where *o*_*GC*_(*g*) is the count of Guanine and Cytosine in gene *g*, and *L*(*g*) is the length of gene *g* counted by nucleotide.

### Distance to HEG and HEG probability

In 2011, Menzella’s research suggested that replacing all codons by the most preferable codons could lead to an inferior yield because of an imbalanced tRNA pool. Additionally, a low concentration of favorite usage codons also causes decrease in the translational level. In this case, estimating the most appropriate CAI value is an unlikely task [11, 22]. Hence, we proposed two novel features called HEG probability (HEGP) and distance to HEG (DHEG).

Given a gene *g*, the event that *g* is a member of *HEG* set or not is a random variable. The probability that *g* belongs to the reference set of *HEG* is shown as (5)

5 $$ P\left( HEG\Big| g\right)=\frac{P\left( g\Big| HEG\right) P(HEG)}{P\left( g\Big| HEG\right)+ P\left( g\Big|\overline{HEG}\right)} $$

6 $$ P\left( g\Big| HEG\right)= P(HEG)\times {\displaystyle \prod_{c\in C}}\frac{e^{-\frac{{\left({r}_{ac}(g)-{\mu}_c\right)}^2}{2{\sigma}_c^2}}}{\sigma_c\sqrt{2\pi}} $$

7 $$ P\left( g\Big|\overline{HEG}\right)= P\left(\overline{HEG}\right)\times {\displaystyle \prod_{c\in C}}\frac{e^{-\frac{{\left({r}_{ac}(g)-{\overline{\mu}}_c\right)}^2}{2{\overline{\sigma}}_c^2}}}{{\overline{\sigma}}_c\sqrt{2\pi}} $$

where *c* is a codon in a set of possible codon *C*, $\overline{HEG}$ is a non-highly expressed genes set, *μ*_*c*_ $\left({\overline{\mu}}_c\right)$ and *σ*_*c*_ $\left({\overline{\sigma}}_c\right)$ is the mean and standard deviation of *r*_*ac*_ of all genes in *HEG* set ($\overline{HEG}$ set, respectively). In some cases that $P\left( g\Big|\overline{HEG}\right)$ is much smaller than *P*(*g*|*HEG*), *P*(*HEG*|*g*) can be high although *g* is too different from *HEG* and $P\left( g\Big|\overline{HEG}\right)$ is low. In order to limit this situation, we defined a new Eq. (8) limited by principle components analysis (PCA) [37, 38]

8 $$ {P}_{final}\left( HEG\Big| g\right)=\left\{\begin{array}{c}\hfill P\left( HEG\Big| g\right),\kern0.5em {p}_i(g)\in \left[ \min {p}_i\left({g}_{HEG}\right), \max {p}_i\left({g}_{HEG}\right)\right]\hfill \\ {}\hfill 0,\kern0.5em  otherwise\hfill \end{array}\right. $$

where *p*_*i*_(*g*) is the *i*_¬_^th^ principle component of vector (*r*_*a*1_(*g*), …, *r*_*a*59_(*g*))^*T*^, *g*_*HEG*_ is a gene in *HEG* set, and $i=\overline{1,2}$. We only used two principle components, thus, we could observe HEGP on a 2-dimensional surface.

Resembling HEGP, DHEG was used to calculate the similarity between the candidate gene *g* and the reference set as (9). We normalized DHEG by scaling *D*(*g*, *HEG*) to [0,1] such that *D*(*g*, *HEG*) = 0 if *g* and *HEG* are totally different, and *D*(*g*, *HEG*) = 1 if they highly resemble, see (10). In our experiment, $\underset{g}{ \min } D\left( g, HEG\right)=4$ and $\underset{g}{ \max } D\left( g, HEG\right)=17$.

9 $$ D\left( g, HEG\right)=\frac{1}{\left| HEG\right|}\sum_{g_{HEG}\in HEG}{\left\Vert \left(\begin{array}{c}\hfill {r}_{a1}(g)\hfill \\ {}\hfill \vdots \hfill \\ {}\hfill {r}_{a59}(g)\hfill \end{array}\right)-\left(\begin{array}{c}\hfill {r}_{a1}\left({g}_{HEG}\right)\hfill \\ {}\hfill \vdots \hfill \\ {}\hfill {r}_{a59}\left({g}_{HEG}\right)\hfill \end{array}\right)\right\Vert}_2 $$

10 $$ {D}_{final}\left( g, HEG\right)=\frac{D\left( g, HEG\right) - \underset{g}{ \min } D\left( g, HEG\right)}{\underset{g}{ \max } D\left( g, HEG\right)-\underset{g}{ \min } D\left( g, HEG\right)} $$

**Fig. 1:**
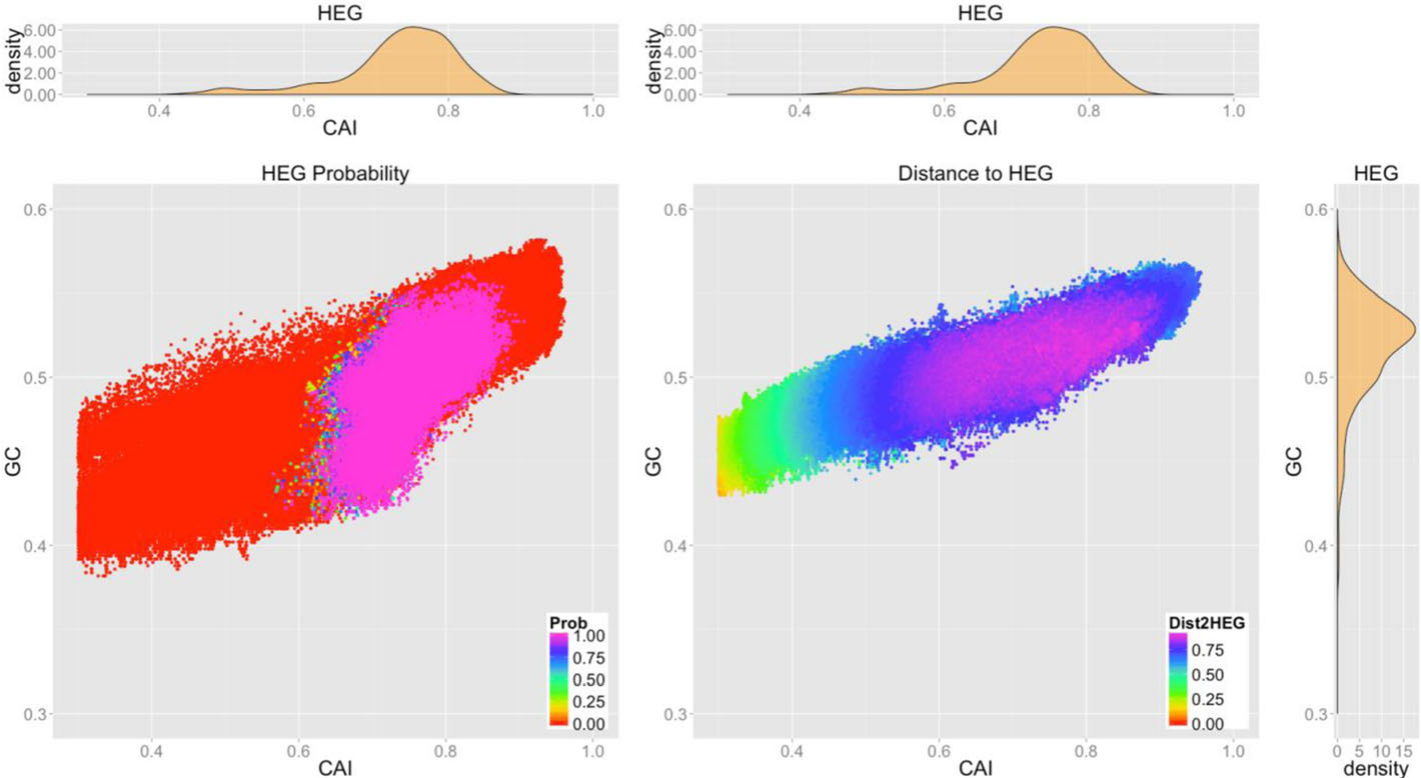
Properties of HEGP and DHEG. The top plots are distribution of HEG’s CAI value. The bottom right plot is distribution of HEG’s GC value. The bottom center plots illustrate HEG probability and distance to HEG of randomly generated gene sequences with respect to their CAI and GC value

In order to investigate the properties of the novel features, we used a genetic algorithm to optimize genes *g* coding for random proteins as (11) or (12)

11 $$ g= \arg \underset{g}{ \max}\left[\left| CAI(g)- i\right|+\left| GC(g)- j\right|+\left|{P}_{final}\left( HEG\Big| g\right)- k\right|\ \right] $$

12 $$ g= \arg \underset{g}{ \max}\left[\left| CAI(g)- i\right|+\left| GC(g)- j\right|+\left|{D}_{final}\left( g, HEG\right)- k\right|\right] $$

where *i*, *j*, *k* ∈ {0.00, 0.01, …, 1.00}. We would like to obtain all possible value of HEGP and DHEG with respect to each pair of CAI and GC value within this process and analyze the association between CAI and GC and HEGP (or DHEG). The results are shown in Results and Discussion and Fig. 1.

### Neural network

We proposed a novel method to construct fitness function for genetic algorithm (in next step) based on neural network (NN), CAI and GC content. A 2-hidden layer network is computed as (17), such that ∑_*g*_|*o*(*g*) − *y*_*g*_|^2^ was minimized, where *y*_*g*_ is the yield of gene *g* collected from experimental data (in Data collection), *m* is the number of nodes at the first hidden layer, and *n* is the number of nodes at the second hidden layer [39]. We estimated $m=\sqrt{3 N}+2\sqrt{\frac{N}{3}}$ and $n=2\sqrt{\frac{N}{3}}$ as Huang suggested in 2003, where *N* is number of samples in data set [40].

13 $$ {o}_1(g)=\left(\begin{array}{ccc}\hfill {w}_{1,1}^1\hfill & \hfill \cdots \hfill & \hfill {w}_{1,3}^1\hfill \\ {}\hfill \vdots \hfill & \hfill \ddots \hfill & \hfill \vdots \hfill \\ {}\hfill {w}_{m,1}^1\hfill & \hfill \cdots \hfill & \hfill {w}_{m,3}^1\hfill \end{array}\right)\times \left(\begin{array}{c}\hfill CAI(g)\hfill \\ {}\hfill GC(g)\hfill \\ {}\hfill 1\hfill \end{array}\right) $$

14 $$ {h}_1(g)=\frac{1}{1+{e}^{-{o}_1}} $$

15 $$ {o}_2(g)=\left(\begin{array}{ccc}\hfill {w}_{1,1}^2\hfill & \hfill \cdots \hfill & \hfill {w}_{1, m+1}^2\hfill \\ {}\hfill \vdots \hfill & \hfill \ddots \hfill & \hfill \vdots \hfill \\ {}\hfill {w}_{n,1}^2\hfill & \hfill \cdots \hfill & \hfill {w}_{n, m+1}^2\hfill \end{array}\right)\times \left(\begin{array}{c}\hfill {h}_1\hfill \\ {}\hfill 1\hfill \end{array}\right) $$

16 $$ {h}_2(g)=\frac{1}{1+{e}^{-{o}_2}} $$

17 $$ o(g)=\left(\begin{array}{ccc}\hfill {w}_1\hfill & \hfill \cdots \hfill & \hfill {w}_{n+1}\hfill \end{array}\right)\times \left(\begin{array}{c}\hfill {h}_2\hfill \\ {}\hfill 1\hfill \end{array}\right) $$

For the purpose of testing performance of this method, we randomly separated data into 3 parts which were 30% of data for testing, 70% × 30% = 21% of data for validation, and 70% × 70% = 49% of data for training. Training, validation, and testing processed were repeated 100 times to reduce impact of over fitting, and the final model was an arithmetic mean of these 100 NNs, (18).

18 $$ {o}_{final}=\frac{1}{100}{\sum}_{i=1}^{100}{o}^{(i)}(g) $$

We also restricted NN by HEGP (or DHEG) such that *o*_*final*_ = *P*_*final*_(*HEG*|*g*) (or *o*_*final*_ = *D*_*final*_(*g*, *HEG*)) if c (or *D*_*final*_(*g*, *HEG*) < 0.75), otherwise *o*_*final*_ is considered as (18). These were called NN restricted by HEGP (NNP) and NN restricted by DHEG (NND).

### Multivariable linear regression

Linear functions were commonly used in gene optimization [18, 27], as (19). In this study, we proposed estimating parameters such that ${\sum}_g{\left({y}_g-\widehat{y_g}\right)}^2$ was minimized [41]. We also separated data as Neural network for comparison purposes and the final model was constructed by using whole data set.

19 $$ \widehat{y_g}=\left(\begin{array}{cc}\hfill \widehat{w_1}\hfill & \hfill \widehat{w_2}\hfill \end{array}\right)\times \left(\begin{array}{c}\hfill CAI(g)\hfill \\ {}\hfill GC(g)\hfill \end{array}\right)+\hat{\mathit{\in}} $$

### Genetic algorithm

The genetic algorithm which was inspired by natural selection and evolution processes is naturally appropriate to the gene optimization task in that each gene was assigned as a chromosome or an individual [6, 42, 43]. These genes could be evaluated by a fitness function which are *P*_*final*_(*HEG*|*g*), *D*_*final*_(*g*, *HEG*), *o*_*final*_(*g*) or $\widehat{y_g}$. Generation to generation, the algorithm would converge and reach the maximum value of fitness function. Finally, we found the best gene with respect to the fitness function.

## Results and Discussion

### Properties of HEGP and DHEG

Figure 1 illustrates the distributions of HEGP and DHEG in the 2-dimensional vector space constructed by CAI and GC content value. As Fig. 1 described, HEGP of genes varies from 0.00 to 1.00. However, because of the PCA technique, genes having high HEGP tend to cluster together and separate completely from the other genes having minimum HEGP by a discriminant boundary.

DHEG also varies from 0.00 to 0.70, but there is no boundary separating regions of high and low DHEG. Genes having high HEGP or DHEG seem to distribute in the region of high CAI and GC content density. This result suggests that both HEGP and DHEG associate with HEG set in CAI and GC content aspects.

### Properties of NN and comparison between NN and linear regression

**Fig. 2:**
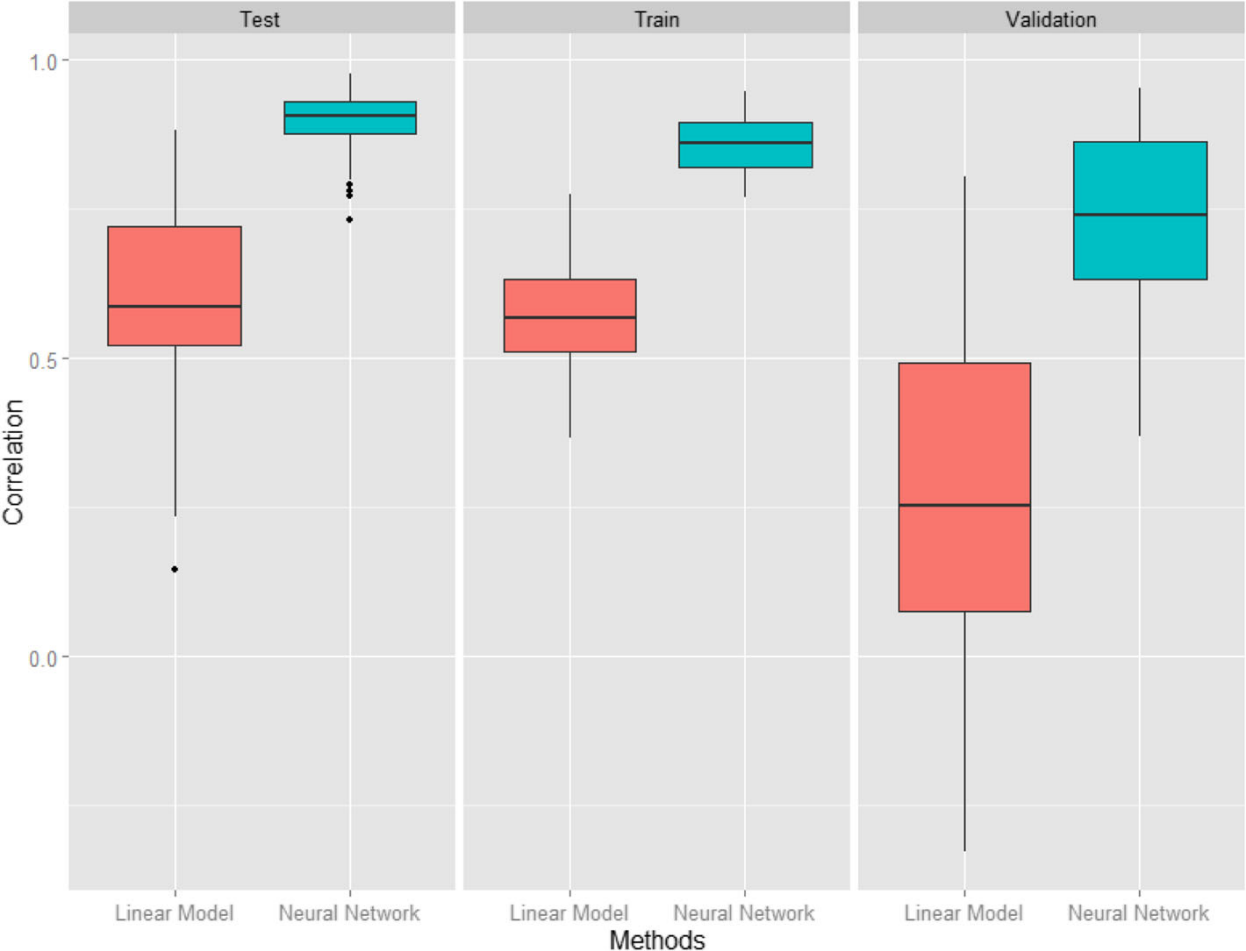
Comparison between NN and linear regression

In comparison with linear regression method, correlation of NN is 1.50, 2.69, and 1.50 times higher than that of linear regression within training, validation, and testing processes, respectively, Fig. 2. A Shapiro-Wilk test shows that almost all data do not fit a normal distribution, so we used a nonparametric Wilcoxon signed-rank test to investigate whether there is any significant difference between the correlation given by NN and linear model, Table 2 [44]. The test indicates that correlation coefficients from NN are significantly higher than that given by linear regression (*P*-*value* <2.2 × 10^−6^ for both three processes) [45]. This result suggests that NN is much more accurate than linear regression. In fact, most of phenomena and processes in nature, especially in life science, associate with non-linear models. For instance, both population growth, gene expression, epidemic spread, etc. models are fitted well with non-linear models. This is a reasonable explanation for the high performance of NN, a non-linear model with sigmoid function.

**Table 2:** *P*-value from Shapiro-Wilk normality test for the correlation of NN and correlation of linear regression

	Training	Validation	Testing
NN	0.03	0.00	9.10 × 10^−5^
Linear regression	0.21	0.11	0.13

**Fig. 3:**
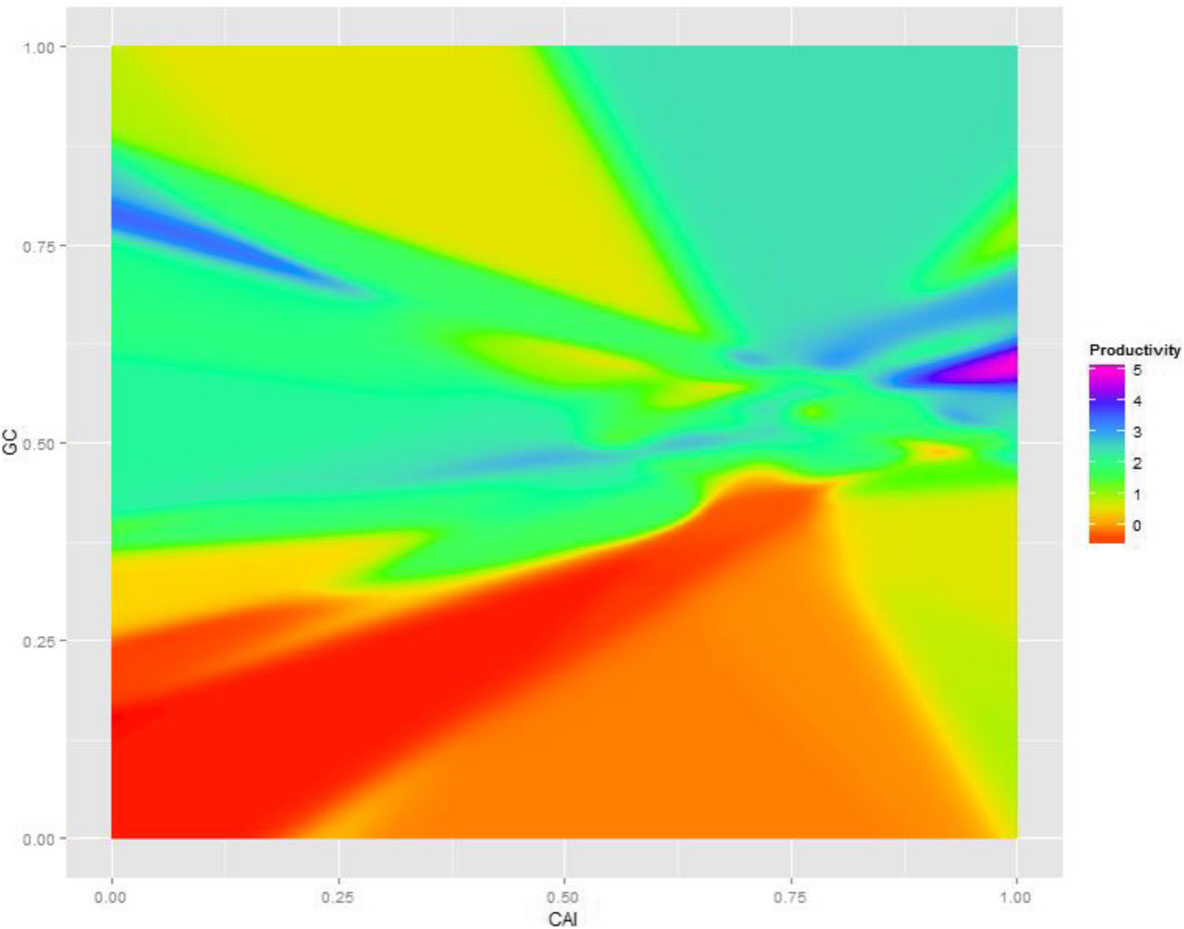
Visualization for fitness function based on NN (log scale) with respect to CAI and GC value

However, NN usually faces an over fitting problem, which causes inaccuracy in practice. As shown in Fig. 3, there are some unreasonable local maximum regions such as the blue region on the top-left corner, and the purple region on the middle-right of the figure. Although genes in these regions have been reported to have low productivity, they still are predicted to have a high translational level. This is the result of a small data set and the complexity of the NN. To overcome this situation, we modified HEGP as in Distance to HEG and HEG probability and the result is shown in Comparison between proposed methods and Application for Escherichia coli to compare between optimization methods.

### Comparison between proposed methods

**Fig. 4:**
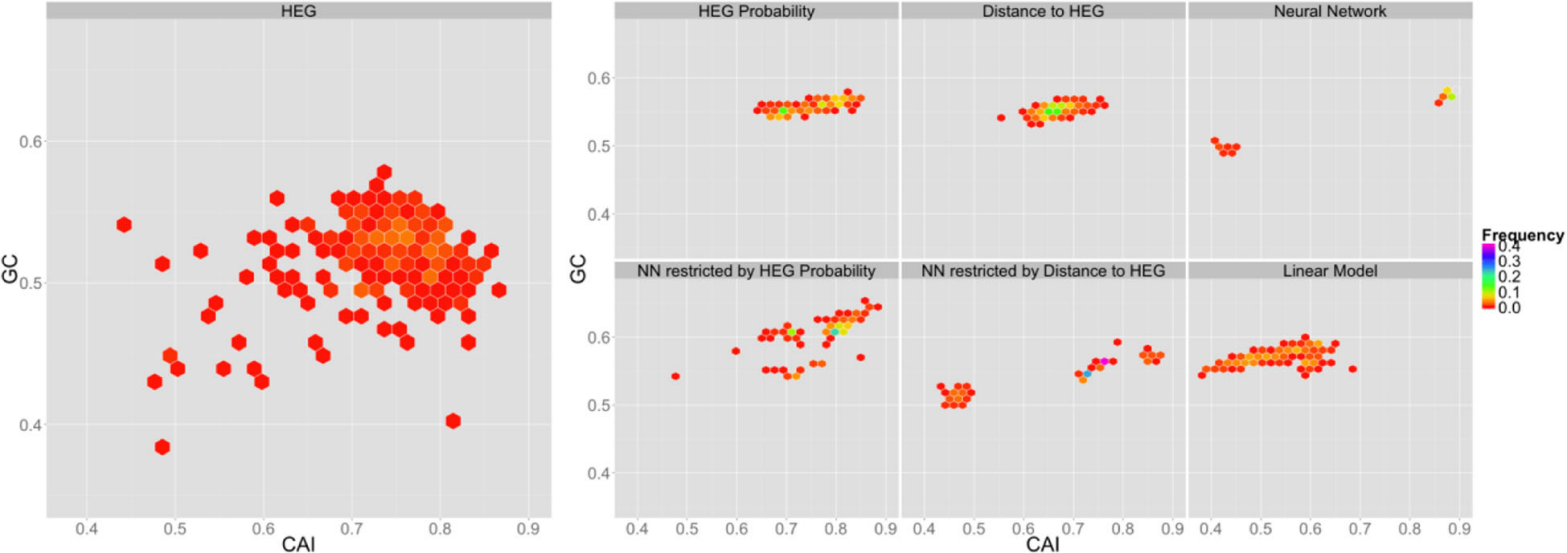
Comparison between gene optimization methods. The plots are 2-dimensional distribution of redesigned genes. X and Y coordinate are CAI and GC value, respectively

We also optimized genes in HEG to compare gene optimization methods and the results are visualized in Fig. 4. While HEGP and DHEG highly appropriate with HEG (differences in descriptive statistics values do not exceed 5% as represented by green cells in Table 3), genes redesigned by linear regression method locate in the region of low CAI (from 0.36 and 0.68) and are quite different from HEG (orange cells in Table 3). NNP is also potential for gene optimization, but NN and NND seem to be unstable and a part of genes optimized by these two methods locate in low CAI region because of the over fitting problem. Both NNP and distances to HEG are the same with HEG, but NN are more than 5% different from HEG. All data in this experiment are not under a normal distribution (Table 4) and the Wilcoxon signed-rank test shows that genes redesigned by HEGP and NND resemble HEG (*P-value* > 0.05), whereas genes optimized by DHEG, NN, NNP and linear model are significantly different from HEG, regarding CAI (Table 5). The distribution of genes designed by NN and linear model are different form HEG so it is reasonable that *P-value* < 0.05 in these cases. Although NNP and DHEG seem to be associated with HEG, the test shows that genes designed by these methods are different from HEG because these methods only focus on high density region of HEG.

**Table 3:**
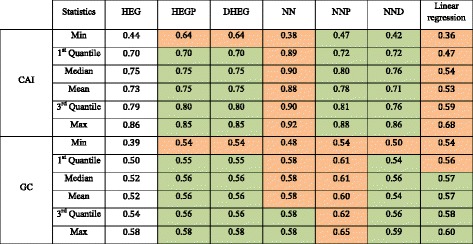
Descriptive statistics for optimized genes

The orange cells represent for values, which are different more than 5% from values of HEG, and vice versa for green cells

**Table 4:**
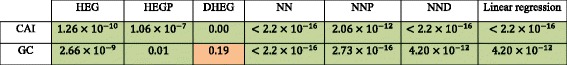
*P*-value from Shapiro-Wilk normality test for optimized genes

**Table 5:**
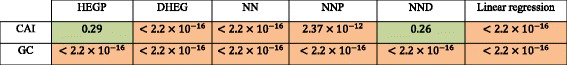
*P*-values from Wilcoxon signed-rank test for difference between HEG and optimized genes

### Application for *Escherichia coli* to compare between optimization methods

We also redesigned gene coding for prochymosin, which was well optimized by Menzella to introduce to *Escherichia coli* in 2011, in order to compare with JCat and EuGene programs [11, 18, 19]. Menzella’s study suggested that CAI of HEG coding for prochymosin are from 0.70 to 0.74 and CAI of the gene giving highest yield is 0.72. Genes having CAI that is out of that range were reported as to be low expressed. In this study, we used the best gene of Menzella’s study as the criteria to evaluate and compare gene optimization method. As Table 6 described, all redesigned genes give the same GC content as the one of Menzella. Only DHEG gives CAI in highly expressed range (0.73) and the CAI value is just lower than CAI of the standard genes by 1.39%, whereas the ones from NN are 34.72% lower than the criteria of CAI. CAI from JCat, EuGene, and linear model are considerably different from the standard by 33.33, 30.56, and 27.78%, respectivly. There are just slightly differences, which are 12.50, 6.94, and 11.11% between the best gene from Menzella’s study and genes optimized by HEGP, NNP, and NND. Gene redesigned by NND is most matched with Menzella’s gene (311), while EuGene gives the worst (67). Matching results of other methods are roughly the same, from 249 to 291. According to these results, we can indicate that EuGene and NN seem not to appropriate to redesign prochymochin. Although matching result from JCat is the fourth highest (280), JCat is also inappropriate because of the high CAI value (0.96). DHEG is likely the most appropriated method with reasonable CAI (0.73) and high matching result (291). HEGP, NNP, and NND give CAI values, which are slightly different from Menzella’s result, but these are also potential methods because genes optimized by these methods highly match with the best gene of Menzella.

**Table 6:** Result of optization for gene coding for prochymosin and comparison with experimental result from Menzella’s study

Method	CAI	GC	Patterns matching				
Menzella	0.72	0.49	6 nucleotides	7 nucleotides	8 nucleotides	9 nucleotides	Total
Jcat	0.96	0.50	177	69	22	12	280
Eugene	0.94	0.50	48	20	6	2	76
HEGP	0.81	0.51	173	55	15	6	249
DHEG	0.73	0.50	216	61	13	1	291
NN	0.47	0.49	185	57	20	7	269
NNP	0.67	0.50	204	68	26	13	311
NND	0.64	0.50	198	63	17	4	282
Linear	0.52	0.52	185	64	13	2	264

**Fig. 5:**
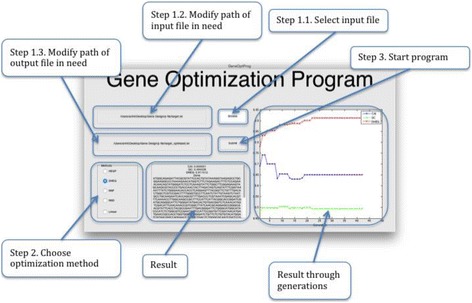
Demonstration program and user’s guide

Finally, we built a demonstrative program using Matlab R2014a under Mac OS X. The program was published in both standalone executable program and Matlab function files. As in Fig. 5, gene optimization includes 3 steps:

- Step 1. Select target protein sequences in FASTA format
- Step 2. Choose optimization method
- Step 3. Start program.

While the program run, the text box on the bottom and the chart on the right will illustrate the progress. The result will be presented in the text box and also stored as FASTA format. The developed program can be downloaded from http://www.math.hcmus.edu.vn/~ptbao/paper_soft/GeneOptProg/.

There are limitations of our study. Firstly, HEG reference set for CAI computing is obtained from predictive method with no laboratory evidence showing that the set is actual HEG dataset. Other studies share the same problem, but the redesigned gene based on CAI computation with predicted HEG are highly expressed [19, 29–31]. Secondly, NN is sensitive in that a small change of input can lead to a significant change of output and it also tends to over fit the training data. Data is collected from different sources with a limited number of samples under variety of experimental environments. These contributes to over-fitting of the NN method. Lastly, although the optimized genes closely resemble highly expressed redesigned genes in related studies, results of proposed methods are not verified by wet lab experiments.

## Conclusions

In this study, we proposed the uses of HEGP, DHEG, and NN to optimize genes and also indicated an approach to estimate parameters for linear function in gene optimization. The correlations of our proposed NN method are from 1.5 to 2.69 times greater than these of linear regression method. Additionally, genes redesigned by the proposed methods associate with HEG whereas genes optimized by popular linear function give low CAI. Therefore, it is concluded that our proposed methods can be potential for gene optimization and further research in gene expression.

In the future, more redesigned genes will be collected to enrich our database to improve the performance of NN. In addition, a mathematical model based on differential equation will be developed to investigate how codon usage bias and tRNA concentration influence translation expression level. The developed model can then be applied in gene optimization. Finally, experiment will be carried out to test the proposed methods and hypothesis.

## Abbreviations

*C*_*a*_: All different types of codon encoding for a specific amino acid *a*
*D*_*final*_(*g*, *HEG*): Normalized distance between gene *g* and HEG
*P*_*final*_(*HEG*|*g*): Probability that *g* belongs to the reference set of *HEG*, limited by PCA
*k*_*a*_ = |*C*_*a*_|: Number of different types of codon encoding for a amino acid *a*
*o*_*GC*_(*g*): Count of Guanine and Cytosine in gene *g*
*o*_*ac*_(*HEG*): Count of codon *c* coding for amino acid *a* in HEG set
*o*_*final*_: Average output of 100 NNs
*r*_*ac*_(*g*): RSCU for codon *c* coding for amino acid *a*
*w*_*ac*_: Relative adaptiveness of codon *c* coding for amino acid *a*
CAI: Codon adaptation index
DHEG: Distance to HEG
HEG: Highly expressed genes/gene
HEGP: HEG probability
NN: Neural network
NND: NN restricted by DHEG
NNP: NN restricted by HEGP
PCA: Principle components analysis
RSCU: Relative synonymous codon usage
*D*(*g*, *HEG*): Distance between gene *g* and HEG
*GC*(*g*): GC content of gene *g*
*L*(*g*): Length of gene *g* counted by codon/nucleotide
*P*(*HEG*|*g*): Probability that *g* belongs to the reference set of *HEG*
*g*: A gene
*g*(*i*): The *i* ^th^ codon of gene *g*
*o*(*g*): Output of NN
